# Superficial stellate cells of the dorsal cochlear nucleus

**DOI:** 10.3389/fncir.2014.00063

**Published:** 2014-06-10

**Authors:** Pierre F. Apostolides, Laurence O. Trussell

**Affiliations:** Oregon Hearing Research Center and Vollum Institute, Oregon Health and Science UniversityPortland, OR, USA

**Keywords:** interneurons, auditory pathways, glycine, gap junctions, electrical synapses

## Abstract

The dorsal cochlear nucleus (DCN) integrates auditory and multisensory signals at the earliest levels of auditory processing. Proposed roles for this region include sound localization in the vertical plane, head orientation to sounds of interest, and suppression of sensitivity to expected sounds. Auditory and non-auditory information streams to the DCN are refined by a remarkably complex array of inhibitory and excitatory interneurons, and the role of each cell type is gaining increasing attention. One inhibitory neuron that has been poorly appreciated to date is the superficial stellate cell. Here we review previous studies and describe new results that reveal the surprisingly rich interactions that this tiny interneuron has with its neighbors, interactions which enable it to respond to both multisensory and auditory afferents.

## Introduction

**Figure 1:**
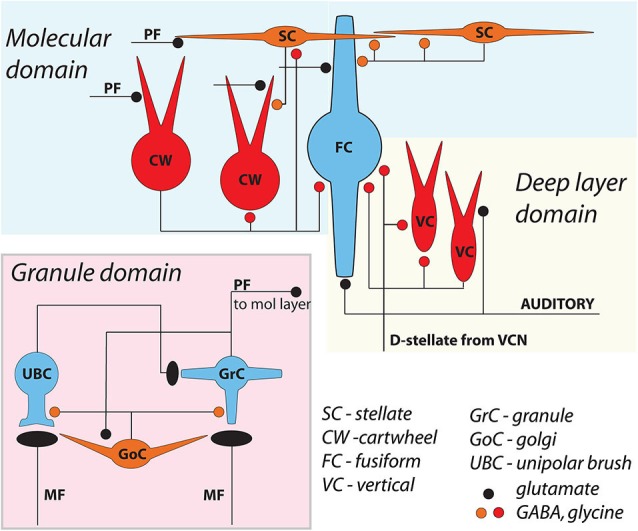
**General circuit diagram of the DCN, divided into three computational domains** The auditory domain comprises the auditory input to fusiform cell basal dendrites, and its modification by vertical and D-stellate interneurons. The non-auditory domain receives mossy fiber input to granule cells, and is modified by Golgi and unipolar brush cells. The molecular layer domain comprises the parallel fiber input from granule cells, terminating on fusiform cell apical dendrites and onto cartwheel and SSC cells, both of which in turn control fusiform activity. Omitted here are the giant cells, whose local synaptic circuitry is not well understood.

The dorsal cochlear nucleus (DCN) is an auditory structure unique to mammals, with anatomical, physiological and molecular similarities to the cerebellar cortex and the electrosensory lobe of mormyrid electric fish (ELL; Oertel and Young, 2004; Bell et al., 2008). Fusiform principal cells receive auditory input onto their basal dendrites and multisensory input onto their apical dendrites (Figure 1). Each type of input signal is preprocessed by a system of interneurons. The function of some of these interneurons (tuberculoventral or vertical cell, and the cartwheel cell) have been established through a combination of *in vivo* and *in vitro* studies over many years. Others (granule, Golgi and unipolar brush cell) are currently under study but their basic function may be generally understood by analogy to their counterparts in the cerebellar cortex and ELL.

However, one cell type, the superficial stellate cell (SSC), has received little attention over the years. Several reasons may account for this neglect: SSCs are sparse, tiny cells positioned just under the ependymal cell layer, features that all present challenges for targeting during *in vivo* recordings. However *in vitro* brain slice preparations have recently made it easier to visualize and reach these cells with electrodes, particular in mouse lines in which genetically-encoded fluorophores are expressed in SSCs. Through our studies, several surprising features have come to light about the SSCs that inspire a renewed effort to understand the function of these neurons. Indeed, while in some ways homologous to cerebellar molecular layer stellate cells, SSCs exhibit properties that place them in a computationally unique position in the entire cochlear nucleus. Their size heightens their sensitivity to small inputs and their location optimizes their ability to communicate with specific dendrites of DCN principal cells. Most interestingly, gap junctions in SSCs are used to communicate both excitatory and inhibitory signals between the auditory and multisensory domains.

## Methods

**Figure 2:**
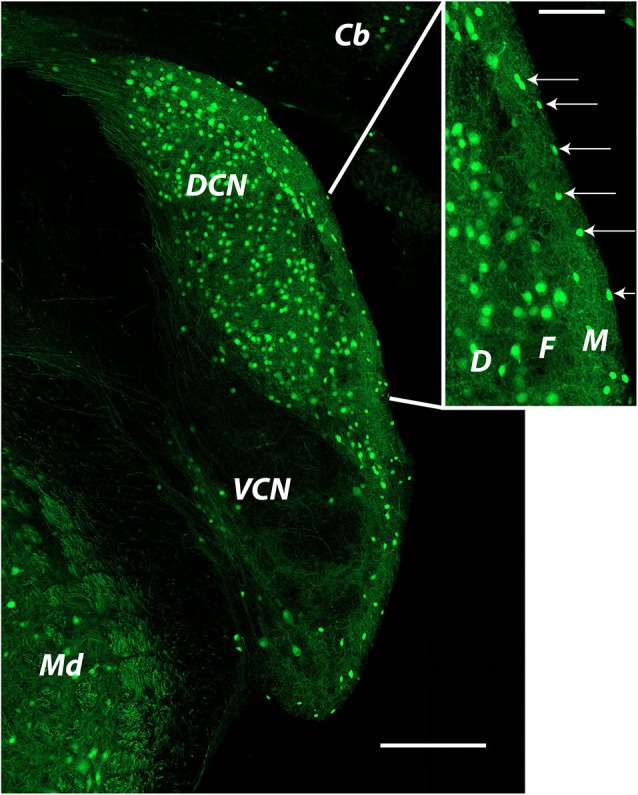
**Distribution of glycinergic neurons in the cochlear nucleus as revealed by GFP labeling in a GlyT2-GFP mouse** The image was produced from tiled images captured at a single focal plane with a 20x objective. Cells identified as SSCs (arrows in inset) were small bright cells located in the DCN molecular layer. Cells used for recordings were most often those closest to the edge of the brain stem at the ependymal layer. Cb: cerebellum; DCN: dorsal cochlear nucleus; Md: medulla; VCN: ventral cochlear nucleus. Inset: D: deep layer; F: fusiform cell layer; M: molecular layer.

**Figure 3:**
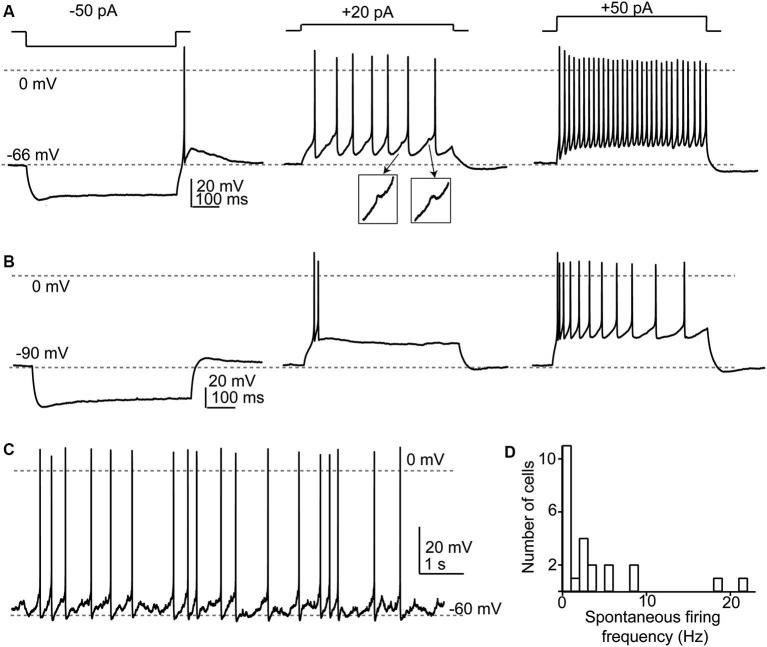
**Response properties of SSCs. (A)** Responses to current injections to three different levels illustrating a regular firing pattern. Note that in the middle panel spikelets (shown in insets) were apparent between the full-amplitude spikes. **(B)** When depolarized from more negative membrane potentials, SSCs generated spike bursts or exhibited an adapting profile of spiking. **(C)** Example of spontaneous spike activity that was apparent in about half of recorded SSCs. **(D)** Broad frequency distribution of spontaneous spiking in a population of 29 SSCs.

Methods are for new data presented in Figures 2, 3, and 6.

**Figure 4:**
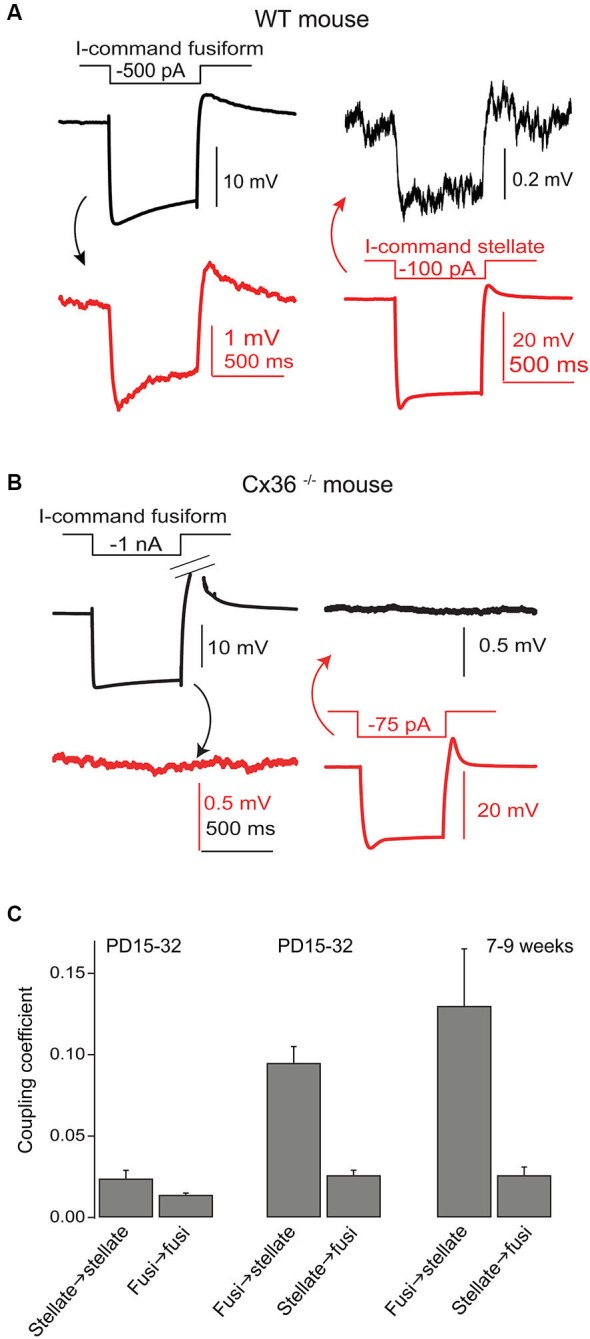
**Gap junctions couple SSCs and fusiform cells. (A)** Coupling between a recorded SSC and fusiform pair was tested by injection of hyperpolarizing currents in one cell and then the other, as indicated. Hyperpolarizations of smaller amplitude in the postjunctional cell was taken as evidence of coupling. **(B)** A similar experiment was performed in a Cx36 knockout mouse, and showed no evidence of coupling. **(C)** Average coupling coefficients reveal bias for transmission from fusiform cell to SSC. This coupling was present in mice up to 9 weeks postnatal. Data adapted from Apostolides and Trussell (2013b).

**Figure 5:**
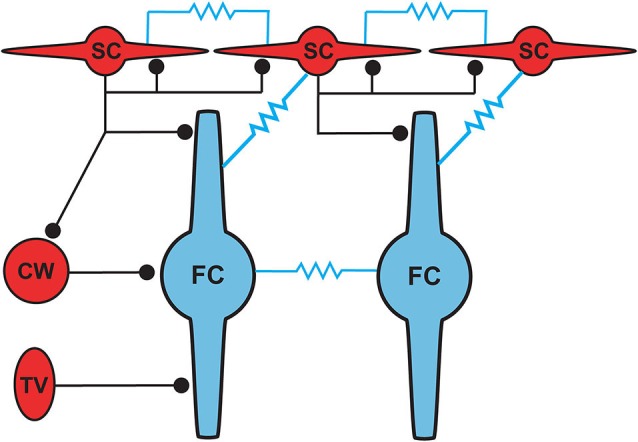
**Proposed circuit diagram for inhibitory inputs to fusiform cells and the gap junction connectivity (represented by resistors) between SSCs and fusiform cells**. SSCs, cartwheel and tuberculoventral cells occupy distinct domains of the fusiform somatodendritic space. SSCs are unique among the three inhibitory cell types in their additional electrical connectivity with fusiform cells and with one another.

**Figure 6:**
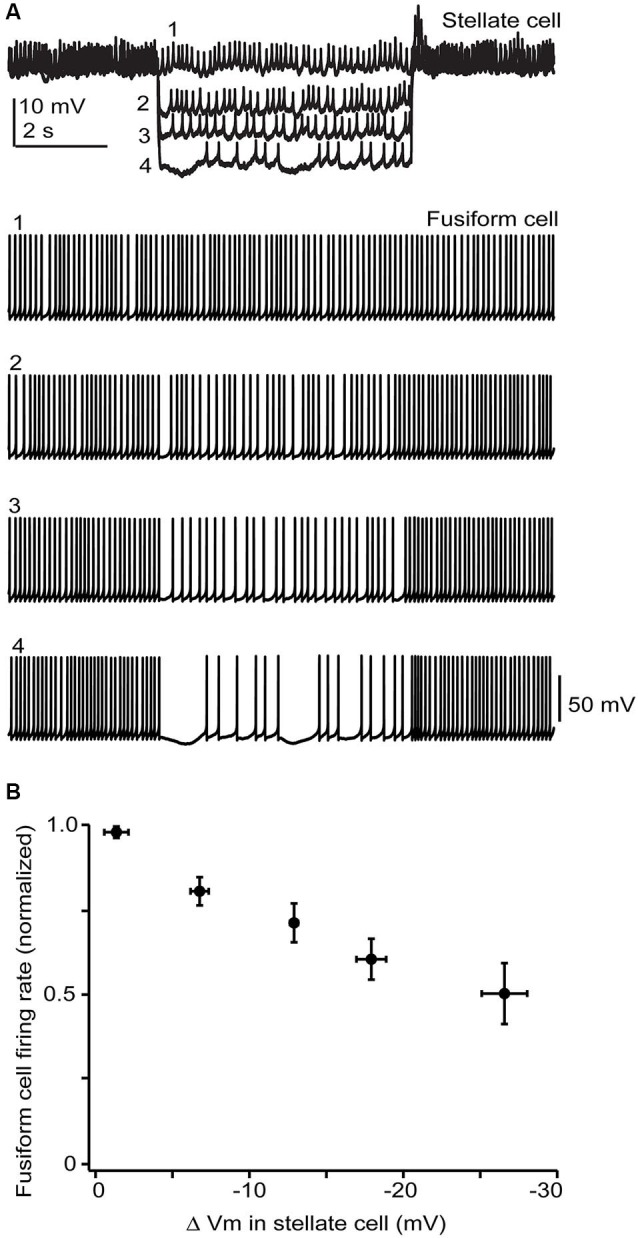
**Stellate membrane potential modulates fusiform spontaneous spike rate. (A)** In an electrically coupled cell pair, injection of hyperpolarizing current steps into the SSC shifted the SSC potential up to 20 mV negative to the resting potential of −65 mV and had a clear inhibitory effect on fusiform spontaneous spiking. **(B)** Average data from 5 cell pairs shows a near linear dependence of fusiform cell spike rate on SSC membrane potential. Firing rates varied widely among fusiform cells and so were normalized in each cell to the rate during the baseline condition where no hyperpolarizing current step was injected into the stellate cell.

### Slice Preparation

Experimental procedures were approved by OHSU’s Institutional Animal Care and Use Committee. C57/Bl6 mice P15-P24 were anesthetized with isofluorane, decapitated, and slices (200–250 µm thick) containing the DCN were cut in an ice-cold sucrose solution which contained (in mM): 87 NaCl, 25 NaHCO_3_, 25 glucose, 75 sucrose, 2.5 KCl, 1.25 NaH_2_PO_4_, 0.5 CaCl_2_, 7 MgCl_2_, bubbled with 5% CO_2_/95% O_2_. Slices subsequently recovered for 30–45 min at 34°C in artificial cerebrospinal fluid (ACSF) solution which contained (in mM): 130 NaCl, 2.1 KCl, 1.7 CaCl_2_, 1 MgSO_4_, 1.2 KH_2_PO_4_, 20 NaHCO_3_, 3 Na-HEPES, 10–12 glucose, bubbled with 5% CO_2_/95% O_2_ (300–310 mOsm). This solution was also used as the standard perfusate for all experiments. In some experiments 5 µM 3-((R)-2-Carboxypiperazin-4-yl)-propyl-1-phosphonic acid (R-CPP) or 50 µM D-2-amino-5-phosphonovalerate (D-APV) were added to the cutting solution and/or recovery chamber. After recovery, slices were maintained at 22°C until recording, typically within 5 h of slice preparation.

### Electrophysiology

Slices mounted in the recording chamber were continuously perfused at 3–5 ml/min with ACSF (31–33°C) and visualized using Dodt contrast optics using either a 40x or 63x objective on a Zeiss Axioskcop 2 microscope. Patch pipette solution contained (in mM) 113 K-gluconate, 4.8 MgCl_2_, 4 ATP, 0.5 GTP, 10 Tris-phosphocreatine, 0.1–0.2 EGTA, 10 HEPES, pH adjusted between 7.2–7.3 with KOH (∼290 mOsm). Pipette resistances for fusiform and stellate cells were typically 2–3 and 3–5 MOhm, respectively, when filled with the K-gluconate solution. Pipette capacitance was cancelled and series resistance effects adjusted with bridge balance.

### Cell identification

Stellate cells were identified by their small size and location in the slice at the outer edge of the molecular layer just below the ependymal surface. In experiments with transgenic mice, GFP fluorescence was observed with a 100 W Hg bulb placed in the epi-fluorescence port of the microscope and passed through a GFP filter. Fusiform neurons were identified as large cells situated in the DCN cell body layer, and showed spike characteristics as previously described (Zhang and Oertel, 1993; Golding and Oertel, 1997).

### Data Acquisition and Analysis

Data were recorded with a Multiclamp 700B amplifier and a Digidata 1322A analog-digital converter board using pClamp 9 software. Signals were low-pass filtered at 10–20 kHz and digitized at 20–50 kHz. Data were analyzed offline after filtering the traces at 2–10 kHz. All values are reported as mean ± SEM.

### GlyT2-GFP mice

Recordings were initially made from mice expressing GFP under the control of the promoter for the neuronal glycine transporter GlyT2 (Zeilhofer et al., 2005), to aid in learning to identify SSCs in our slices. To examine the distribution of this GFP label among cells in the cochlear nucleus, mice were transcardially perfused with warm (38°C) 100 mM phosphate buffered saline (PBS) solution, pH 7.4, followed by ice-cold 4% paraformaldehyde in PBS. The brains were dissected from the skull and incubated overnight in 4% paraformaldehyde for complete tissue fixation. Brains were rinsed in PBS and coronal sections were cut at 30 µm using a vibratome. The sections were washed in PBS solution for 30 min and then slide mounted and coverslipped in Fluoromount G medium (Southern Biotechnology Associates).

### Reagents

2,3-Dioxo-6-nitro-1,2,3,4-tetrahydrobenzo[f]quinoxaline-7-sulfonamide (NBQX), APV, CPP, SR95531 were purchased from Ascent Scientific/Abcam. Strychnine was purchased from Sigma-Aldrich.

## Results

### Previous studies

Cells resembling SSCs in the DCN have been described in anatomical studies stretching back over a century (Cajal, 1911; Brawer et al., 1974; Kane, 1974; Disterhoft et al., 1980; Nó, 1981; Webster and Trune, 1982). However, it was not until the landmark Golgi and electron microscopy (EM) study of Wouterlood et al. (1984) that we had a comprehensive approach specifically to the SSCs. Those authors described small cells whose soma, dendrites and axons were restricted to the outermost, molecular layer of the DCN, and in this sense resembled the stellate cells of the molecular layer of the cerebellar cortex. Ultrastructurally, Gray’s Type II terminals made by SSCs suggested that these cells were inhibitory, a conclusion supported by a subsequent paper showing expression of glutamic acid decarboxylase (GAD) in SSCs (Mugnaini, 1985). Terminals of stellate cells were seen on dendrites restricted largely to the DCN molecular layer, dendrites belonging to the fusiform principal cells, cartwheel interneurons, and other SSCs. With this description, a picture emerges of domains of inhibition in the DCN, and most interestingly of subcellular domains of individual fusiform cells. Synapses made by SSCs terminate on apical dendrites, boutons of inhibitory cartwheel cells terminate on the soma and proximal dendrites (Wouterlood et al., 1984; Rubio and Juiz, 2004), and the terminals of inhibitory tuberculoventral (vertical) cells occupy the soma and basal dendrite (Figure 5; Rubio and Juiz, 2004) Thus, the fusiform cells are controlled by three classes of interneuron having partially overlapping domains of inhibition, with the SSC controlling primarily the apical dendrites.

Wouterlood et al. (1984) also described putative excitatory inputs to SSCs, and ascribed these to the *en passant* terminals of the parallel fiber axons of granule cells. Given the diverse, multimodal control of granule cells by mossy fibers, these observations suggest that SSCs are activated predominately by non-auditory rather than auditory fibers. This conclusion, however, is not entirely accurate, as shown in a brain slice study of Zhang and Oertel (1993), who observed EPSPs in a putative SSC following stimulation of the auditory nerve root in the ventral cochlear nucleus, even though auditory nerve fibers do not reach the DCN molecular layer. Moreover, those authors showed that puffs of glutamate in the ventral cochlear nucleus (VCN) also evoked EPSPs in the SSC, suggesting the possibility that excitatory neurons in the VCN, possibly the T-stellate cells, contact SSCs. As discussed in a later section, we suggest an alternative explanation by which auditory nerve activity may excite SSCs in the absence of direct contact from auditory nerve fibers.

### Recent works

In this section we will summarize our recent publications and include some new observations regarding the synaptic and intrinsic properties of SSCs. Studies on mouse SSCs in our lab have focused on cells in the very outermost part of the molecular layer. Such cells are visible in mouse lines in which GFP is expressed under the control of promoters for GAD or for the neuronal plasma membrane glycine transporter GlyT2 (Apostolides and Trussell, 2014a; Figure 2 shows an example). The GlyT2 mouse line in particular reveals the striking abundance of glycinergic neurons in the DCN, and highlights that the SSC appears as the primary interneuron in the outer region of the DCN molecular layer (Figure 2, inset, arrows). In our published works, as well as the new work described below, we have recorded from SSCs in these locations in coronal slices of mouse DCN.

SSCs had membrane input resistances of about 1 GOhm (Apostolides and Trussell, 2013b) and therefore were sensitive to relatively small current injections as compared to cartwheel or fusiform cells. In a set of 29 neurons studied in current clamp, we now find that when cell membrane potentials were held between −55 and −80 mV, hyperpolarizing current injection revealed a small “sag” in membrane potential typically attributed to an I_H_ conductance (Figures 3A,B). When the negative bias current was small (−71 ± 1 mV with −9 ± 2 pA bias), most cells (24/29) tested fired one or several rebound spikes (mean spike number 1.45 ± 0.12) followed by an after-depolarization (Figure 3A). By contrast, with larger negative bias to maintain a more negative voltage (-88 ± 1 mV with −73 ±10 pA bias) this rebound spike behavior was absent (0 of 21 tested cells; Figure 3B). The drive for rebound firing has previously been attributed to I_H_ and/or T-type Ca^2+^ channels (e.g., Aizenman and Linden, 1999; Kopp-Scheinpflug et al., 2011), and suggests that post-inhibitory rebound firing of SSCs depends on membrane potential history. When held between −55 and −70 mV, SSCs fired repetitively upon positive current injection, and could sustain firing at >100 Hz during 150–200 pA current steps (Figure 3A). By contrast, depolarizations from more hyperpolarized levels (Figure 3B) resulted in either spike bursts or an adapting spike pattern. Notably, during evoked or spontaneous spike activity, small, ∼1 mV spikelets, were often visible in the traces (Figure 3A insets), reflecting electrical coupling to neighboring spiking neurons, as described below. With no added bias current, 14/29 (48%) of SSCs tested fired spontaneous action potentials in current clamp with an average frequency of 6.7 ±1.8 Hz (Figures 3C,D).

### Excitatory glutamatergic inputs

Activation of parallel fibers by a stimulus electrode placed in the molecular layer near a voltage clamped SSC resulted in excitatory postsynaptic currents (EPSCs; Apostolides and Trussell, 2014a). Analysis of the kinetics and pharmacology of these AMPA receptor-mediated responses revealed features similar to those of cerebellar stellate cells, in particular submillisecond EPSC decay times and inward rectification of current voltage relations for the glutamate activated channels. These features are consistent with receptors lacking the GluR2 subunit and therefore having a high Ca^2+^ permeability (Hume et al., 1991; Burnashev et al., 1992) and are quite distinct from the properties of AMPA receptors at parallel fiber synapses onto cartwheel and fusiform cells (Gardner et al., 1999, 2001). Thus, if we assume that parallel fibers constitute a uniform population, postsynaptic receptor subtype is not dictated by the identity of the presynaptic neuron, as has been proposed for auditory nerve targets (Gardner et al., 1999, 2001). Interestingly, the similarities to cerebellar stellate cells suggests that excitatory synapses onto SSCs may exhibit long-term synaptic plasticity (Liu and Cull-Candy, 2000). The possibility of plasticity at these synapses is also hinted at by a distinct difference from cerebellar stellate cells in the expression of NMDA receptors. In the cerebellum, NMDA receptors onto stellate cells appear to be expressed extrasynaptically, and activated only when multiple parallel fibers are fired or when they are fired at high rates (Clark and Cull-Candy, 2002; Nahir and Jahr, 2013). However, single action potentials in single parallel fibers are sufficient to activate NMDA receptors on SSCs (Apostolides and Trussell, 2014a); thus there are two synaptic sources of intracellular Ca^2+^ to SSCs, AMPA and NMDA receptors. It will be of interest to test whether activation of parallel fiber synapses can trigger long-term plasticity, as has been shown at synapses onto cartwheel cells and fusiform cells (Tzounopoulos et al., 2004).

### Inhibitory synapses

As predicted by the studies from Mugnaini and colleagues (Wouterlood et al., 1984; Mugnaini, 1985), we found that SSCs make GABAergic synapses onto cartwheel cells, fusiform cells, onto other SSCs, and even autaptic contacts onto themselves (Apostolides and Trussell, 2013b, 2014a). However, it was clear that these same synapses also released glycine, because an antagonist of GABA_A_ receptors, SR95531, did not fully eliminate inhibitory transmission, but transmission was blocked by a mixture of SR95531 and strychnine. Such co-release and co-transmission is common in auditory brainstem interneurons, but varies according to cell type, possibly due to differential receptor distribution (Dugué et al., 2005; Lu et al., 2008; Apostolides and Trussell, 2013a). We examined co-transmission quantitatively by activating autaptic connections and found that 70% of the IPSC was blocked by SR95531 and the remainder by the glycine receptor antagonist strychnine (Apostolides and Trussell, 2014a). This was then confirmed in experiments in which nearby SSCs were selectively excited and the IPSCs those SSCs then made onto a recorded SSC were analyzed pharmacologically. This approach avoided the potential problem of dialysis of GABA during presynaptic recordings which might otherwise diminish the magnitude of GABAergic transmission (Apostolides and Trussell, 2013a). As the IPSC components produced by the two transmitters had distinct kinetic signatures, the results imply that co-transmission might enable both fast and slow phases of inhibition.

### Gap junction coupling

Wouterlood et al. (1984) predicted on the basis of ultrastructural evidence that gap junctions may form between SSCs, again in alignment with observations from cerebellar stellate cells (Sotelo and Llinas, 1972; Mann-Metzer and Yarom, 1999). In Apostolides and Trussell (2013b) we therefore searched for electrical coupling between cells by making paired recordings between adjacent SSCs in 2–4 week-old mice, and found that indeed voltage displacements in one SSC led to voltage changes in neighboring SSCs in 21% of pairs, with coupling coefficients (the ratio of postjunctional to prejunctional response) of a few percent (Apostolides and Trussell, 2013b). More frequent coupling with similar strength was observed between adjacent fusiform neurons (71% of pairs). However, most striking was the observation that SSCs were also electrically coupled to fusiform cells (45% of pairs), with an apparent preferred direction of communication from principal cell to interneuron (Figures 4A,C). Thus, coupling coefficients for connected pairs were ∼4-fold higher for signals passing from fusiform cells to SSCs than for the reverse direction, and this range of values was maintained in mice at least up to 9 weeks of age (Figure 4C). This developmentally stable, heterotypic electrical connection was blocked by the gap junction blocker meclofenamic acid and was absent in connexin 36 knockout mice (Figure 4B). The basis of the rectification was most likely a simple outcome of “impedance mismatch”, as SSCs had input resistances about ten-fold higher than fusiform cells. Thus, gap junctions between SSCs and fusiform cells facilitate non-chemical, rapid synaptic communication, with a direction that in principle would lead to activation of SSCs when fusiform cells are activated by their parallel fiber or auditory nerve input. Figure 5 summarizes the pattern of electrical contacts observed among fusiform and SSCs, and contrasts these contacts with the pattern of chemical synapses made by SSCs and the other interneurons. In the next sections we will overview what are the functional outcomes of this novel neural pathway.

### Fusiform → SSC transmission

Transmission of biological signals from fusiform to SSCs is evident by the presence of spikelets in SSCs which were shown to originate in fusiform cells (Apostolides and Trussell, 2013b). When spikes in fusiform cells are triggered at high frequency, spikelets in SSCs summated to a low depolarizing plateau. Reasoning that fusiform cells might converge on SSCs, and thus drive them more effectively, two types of experiments were performed. In the first, auditory nerve fibers leading to fusiform cells were activated, and this led to a substantial depolarization of SSCs. This result might at least partially account for the observation of Zhang and Oertel (1993), that stimulation of the nerve produced an apparent EPSP in an SSC. In the second, groups of fusiform cells were activated following light exposure in slices taken from mice expressing channelrhodopsin2 (ChR2) specifically in fusiform cells, which in turn drove action potentials in SSCs.

Three consequences of transmission in this direction were observed. Depolarization of fusiform cells could enhance the potency of depolarizing stimuli delivered directly to SSCs. Moreover, following a period of depolarization, fusiform cells showed a prominent afterhyperpolarization (AHP) which was potently transmitted to SSCs, and could block excitation of SSCs. Finally, spikes triggered in SSCs by fusiform excitation led to synaptic inhibition of all three targets of SSCs: cartwheel cells, neighboring fusiform cells, and other SSCs (Apostolides and Trussell, 2013b, 2014a). Overall, the results suggested the possibility that *in vivo*, SSCs may be driven by auditory nerve activity, not because of direct synaptic input, or even indirect input via mossy fibers and parallel fibers, but rather because auditory signaling in the fusiform cell would be conveyed to the molecular layer through gap junctions.

Besides the modification of SSC firing by fusiform spikes and their AHPs, it was also found that subthreshold EPSPs in fusiform cells are communicated to SSCs, but in a very remarkable manner. In Apostolides and Trussell (2014b), EPSPs generated either by parallel fiber stimulation or by injection of synaptic like current waveforms, were converted to long lasting depolarizations by activation of a subthreshold Na^+^ conductance. This broad EPSP deactivated I_H_, thus leading to an obligatory AHP. The resulting biphasic waveform lasted hundreds of ms and was effectively transmitted to the SSCs through gap junctions. Indeed, given the filtering properties of gap junctions, these results showed that EPSPs in fusiform cells may more effectively modulate the activity of the SSC network than spikes.

### SSC → Fusiform transmission

While the electrical coupling coefficient in the SSC-to-fusiform cell direction was low, we now find that long-lasting hyperpolarizing signals in SSCs may inhibit activity in fusiform cells. Figure 6A shows a recording from an electrically coupled SSC and fusiform cell, as defined by the bi-directional transmission of electrotonic pulses across the two cells (described in Apostolides and Trussell, 2013b). The fusiform cell in this example was spontaneously active, as typical of these cells *in vitro* and *in vivo* (Rhode et al., 1983; Hancock and Voigt, 2002; Leao et al., 2012), and spikelet activity was readily apparent in the adjoining SSC. Negative displacements of the SSC membrane potential from the resting potential had a clear inhibitory effect on the spontaneous firing rate of fusiform cell. Among average data (Figure 6B), spike rate had a nearly linear dependence on SSC membrane voltage between 0 to −30 mV negative to the resting potential. While this effect required relatively large hyperpolarizations, it might be more potent if multiple SSCs were to converge on fusiform cells and were hyperpolarized as a group, perhaps occurring when SSCs receive inhibition from neighboring cells or through the actions of a neuromodulator. In any case, these data complement our previous studies, and show that SSCs inhibit fusiform cells through both chemical and electrical contacts, whereas fusiform cells both excite and inhibit SSCs through electrical contacts.

## Discussion

The network of SSCs in the DCN shares several features with that of stellate cells of the cerebellar cortex, including expression of Ca^2+^-permeable AMPA receptors, formation of GABAergic contacts between stellate cells, autapses, and inhibitory contacts on principal cells (fusiform and Purkinje cells), as well as electrical coupling between stellate cells. But the parallels seem to end there, as SSCs show additional features that are quite distinct from their cerebellar counterparts. Excitatory chemical synapses onto SSCs utilize both AMPA and NMDA receptors, and inhibitory chemical synapses release glycine along with GABA and activate both GABA and glycine receptors. Electrical synapses between stellate cells and Purkinje cells of the cerebellum have not been reported, although one study described dye-coupling between these neurons following exposure to nicotine (Middleton et al., 2008). These and other physiological and molecular differences highlight the different natures of computation in DCN and cerebellum. It will be of interest to contrast the properties of SSCs in these structures with their counterparts in the electrosensory lobe of mormyrid electric fish, which share many of key features with the DCN (Bell et al., 2008).

The positioning of SSCs in the DCN, combined with their unusual synaptic connectivity, suggest interesting potential roles in multisensory integration. Their restriction to the molecular layer, in many cases the very edge of the molecular layer, point to a domain of control limited to the outermost dendritic fields of fusiform and cartwheel cells (Figure 5). Presumably, SSCs have the capacity to suppress excitatory synaptic signals to those dendrites in a region specific manner. As noted above, SSCs thus stand in contrast to the domains of influence of cartwheel and tuberculoventral cells, and this anatomical relationship points to a picture of complex computational subregions in the fusiform cell (Figure 5). Moreover, the observation that cartwheel cells and SSCs contact one another, combined with the possibility of cartwheel-tuberculoventral interconnections (Kuo and Trussell, unpublished observations), suggests that temporal patterns of input to the three interneuronal subtypes could dictate how these subregions of the fusiform cell are recruited and utilized.

Future studies will need to examine several important aspects of SSC function and synaptic topology. Given the precedent of plasticity at cerebellar stellate cell synapses (Liu and Cull-Candy, 2000) and at parallel fiber to cartwheel and fusiform cell synapses (Fujino and Oertel, 2003; Tzounopoulos et al., 2004), it will be of interest to examine use-dependent changes in strength of parallel fiber to SSC contacts. Indeed, given the complex relationships between SSCs and their targets, such plasticity might potently shift the balance between multisensory (via parallel fibers) and auditory nerve (via the fusiform cell and electrical synapses) control of inhibition in the molecular layer.

Just as important is the question of how stellate cells are “hooked up”. Are electrical and chemical contacts random or are there preferential synaptic targets? What is the layout of SSC axons vs. dendrites in relation to fusiform cells, SSCs, and the tonotopic axis along which fusiform cells are distributed? Recent studies have highlighted the concept of “structured connectivity”, and a recent example showed evidence for preferential connectivity among cerebellar stellate cells (Rieubland et al., 2014). In the DCN this question may be particularly interesting given the orthogonal directions of parallel fibers and the tonotopic axis: do gap junctions connect cells receiving common auditory input or common multisensory input? If SSCs form a more broad electrically coupled network, could electrical connections generate synchronized firing among them? If so, then the AHP communicated by the fusiform cells might serve to upset such synchronous firing, as shown for AHPs in cerebellar Golgi cells (Vervaeke et al., 2010). Beyond the SSC itself is the question of whether electrical synapses play a broader role in auditory processing. A likely target for future physiological studies will be the bushy cells of the ventral cochlear nucleus, in which ultrastructural studies have revealed the presence of gap junctions linking adjacent cell bodies and their dendrites (Sotelo et al., 1976; Gómez-Nieto and Rubio, 2009).

Lastly, it will be of important to examine stellate cell function in animal models of tinnitus, a condition characterized by heightened excitability of fusiform cells and the perception of a subjective sound. This has suggested a causal relationship between the two, although it is likely that other cell types and other brain regions are involved. Middleton et al. (2011) used brain slices to show that excitation of cells in the molecular layer generated a flavoprotein autofluorescence image indicative of cell firing. This signal could be enhanced by a GABA_A_ receptor antagonist, suggesting that cell firing was controlled by a local GABAergic input. In noise treated animals that tested positive in a behavioral assay for tinnitus, autofluoresence signals were less well controlled by the antagonist, suggesting that GABAergic inhibition was diminished in the animal model. It is possible that the source of this GABAergic inhibition is the SSC, since the only other inhibitory cell in that region is the cartwheel cell, whose transmission is dominated 80–90% by glycine and glycine receptors (Roberts et al., 2008). Thus, the remarkably rich physiological features of the SSC may warrant a shift in standing from a neglected tiny cell type to a prominent component in multisensory integration and disease.

## Conflict of interest statement

The authors declare that the research was conducted in the absence of any commercial or financial relationships that could be construed as a potential conflict of interest.
